# Bis[*N*,*N*-bis­(diphenyl­phosphan­yl)cyclo­penta­namine-κ^2^ *P*,*P*′]platinum(II) bis­(trifluoro­methane­sulfonate)

**DOI:** 10.1107/S1600536812026359

**Published:** 2012-06-16

**Authors:** Ilana Engelbrecht, Hendrik G. Visser, Andreas Roodt

**Affiliations:** a Department of Chemistry, University of the Free State, PO Box 339, Bloemfontein 9300, South Africa

## Abstract

The title compound, [Pt(C_29_H_29_NP_2_)_2_](CF_3_SO_3_)_2_, consists of a Pt^II^ atom, situated on an inversion centre, coordinated by two diphosphinoamine bidentate ligands and charge-balanced by two trifluoro­methane­sulfonate anions. The Pt^II^ atom has a distorted square-planar geometry defined by the four P atoms. The distortion is illustrated by the P—Pt—P bite angle of 70.31 (4)°. The geometry around the N atom deviates from a trigonal–planar geometry, evidenced by the P—N—P bite angle of 102.3 (2) °. The N atom is displaced by 0.114 (4) Å from the C/P/P plane. In order to coordinate, the orientation of the phenyl rings alters from a *C_s_* conformation to a *C*_2*v*_ conformation. The cyclo­pentane ring is slightly twisted: the puckering parameters are *q*_2_ = 0.420 (5) Å and ϕ = 26.5 (8) °. The trifluoro­methane­sulfonate anion displays a 0.511 (11):0.489 (11) positional disorder. Weak inter- and intra­molecular C—H⋯O hydrogen bonds influence the crystal packing.

## Related literature

For related platinum(II) complexes, see: Farrar & Browning (1995 ▶); Dyson *et al.* (2004 ▶); Cloete *et al.* (2010 ▶); Engelbrecht *et al.* (2010**a* ▶,b* ▶). For diphosphinoamine (PNP) and other *P*-donor ligands, see: Keat *et al.* (1981 ▶); Purcell *et al.* (1995 ▶); Cotton *et al.* (1996 ▶); Otto & Roodt (2001 ▶); Fei *et al.* (2003 ▶); Otto *et al.* (2005 ▶); Muller *et al.* (2008 ▶); Engelbrecht *et al.* (2010**c* ▶,d* ▶, 2011 ▶). For their use in catalytic olefin transformation reactions, see: Haumann *et al.* (2004 ▶); Crous *et al.* (2005 ▶); Booyens *et al.* (2007 ▶); Ferreira *et al.* (2007 ▶). For puckering parameters, see: Cremer & Pople (1975 ▶).
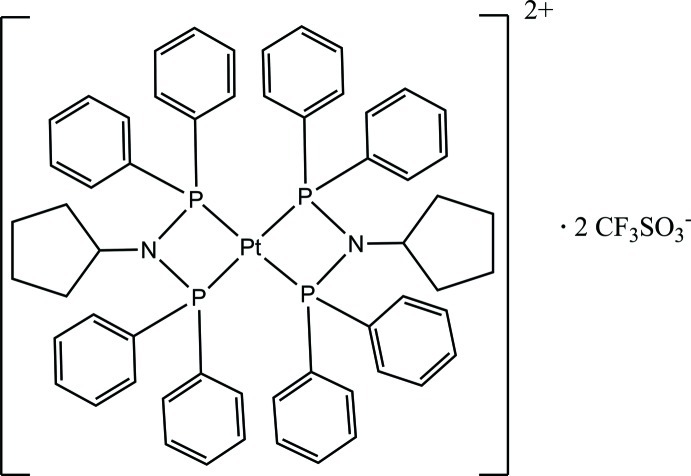


## Experimental

### 

#### Crystal data

[Pt(C_29_H_29_NP_2_)_2_](CF_3_SO_3_)_2_
*M* *_r_* = 1400.18
Monoclinic, 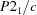

*a* = 10.041 (5) Å
*b* = 13.662 (4) Å
*c* = 20.928 (5) Å
β = 93.916 (5)°
*V* = 2864.2 (19) Å^3^
*Z* = 2
Mo *K*α radiation
μ = 2.71 mm^−1^
*T* = 100 K
0.19 × 0.18 × 0.16 mm

#### Data collection

Bruker APEXII CCD diffractometer
Absorption correction: multi-scan (*SADABS*; Bruker, 2008 ▶) *T*_min_ = 0.627, *T*_max_ = 0.671
44370 measured reflections
6894 independent reflections
4869 reflections with *I* > 2σ(*I*)
*R*_int_ = 0.101

#### Refinement

*R*[*F* ^2^ > 2σ(*F* ^2^)] = 0.040
*wR*(*F* ^2^) = 0.093
*S* = 1.02
6894 reflections
398 parameters
7 restraints
H-atom parameters constrained
Δρ_max_ = 1.39 e Å^−3^
Δρ_min_ = −1.42 e Å^−3^

### 

Data collection: *APEX2* (Bruker, 2011 ▶); cell refinement: *SAINT-Plus* (Bruker, 2008 ▶); data reduction: *SAINT-Plus*; program(s) used to solve structure: *SHELXS97* (Sheldrick, 2008 ▶); program(s) used to refine structure: *SHELXL97* (Sheldrick, 2008 ▶); molecular graphics: *DIAMOND* (Brandenburg & Putz, 2005 ▶); software used to prepare material for publication: *WinGX* (Farrugia, 1999 ▶).

## Supplementary Material

Crystal structure: contains datablock(s) global, I. DOI: 10.1107/S1600536812026359/zb2023sup1.cif

Structure factors: contains datablock(s) I. DOI: 10.1107/S1600536812026359/zb2023Isup2.hkl

Additional supplementary materials:  crystallographic information; 3D view; checkCIF report

## Figures and Tables

**Table 1:** Hydrogen-bond geometry (Å, °)

*D*—H⋯*A*	*D*—H	H⋯*A*	*D*⋯*A*	*D*—H⋯*A*
C25—H25⋯O3*A*	0.95	2.52	3.398 (9)	154
C26—H26⋯O1*B*	0.95	2.5	3.238 (6)	135
C34—H34⋯O3*A* ^i^	0.95	2.38	3.127 (10)	135
C45—H45⋯O3*A* ^ii^	0.95	2.3	3.229 (9)	165
C15—H15⋯O3*A* ^iii^	0.95	2.47	3.420 (11)	178

Symmetry codes: (i) 
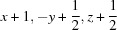
; (ii) 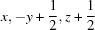; (iii) 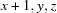.

**Fig. 1.**
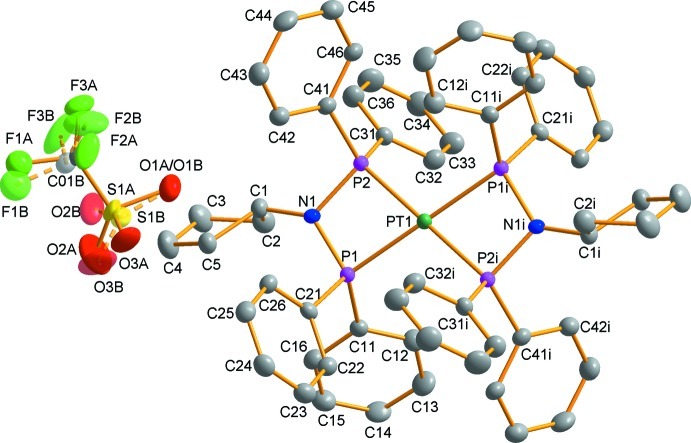
Molecular structure of the title compound. Displacement ellipsoids are drawn at the 50% probability level. Atoms generated through the twofold rotation axis are represented by atoms ending with 'i'. Hydrogen atoms have been omitted for clarity. Dashed lines denote the minor disordered atoms. Symmetry transformations used to generate equivalent atoms: 2 - x, -y, 2 - z.

**Fig. 2.**
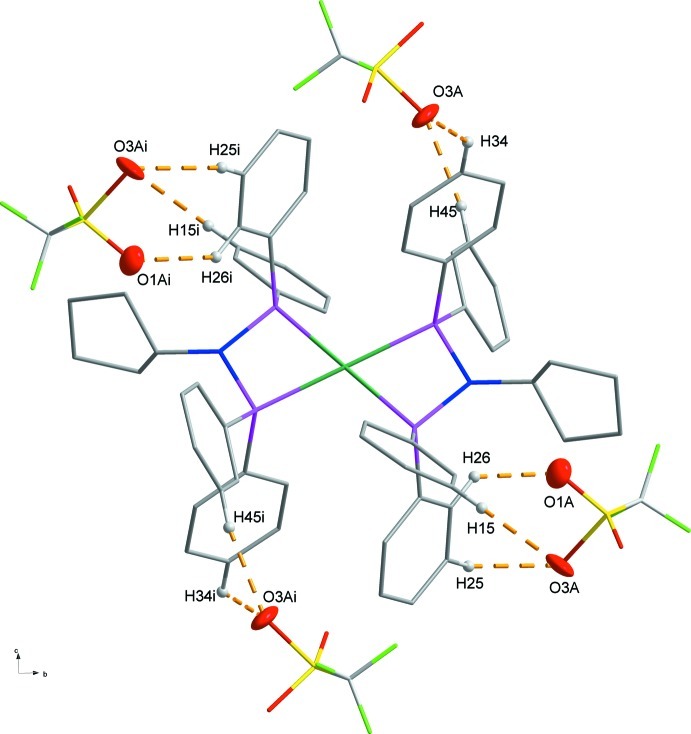
Crystal packing of the title compound, viewed along the a axis showing the hydrogen bonds as dashed lines. Only applicable atoms with relevance to the hydrogen bonds are drawn at the 50% probability level.

## supplementary crystallographic information

### Comment

In the title compound, [Pt(C_29_H_29_NP_2_)_2_](CF_3_SO_3_)_2_, all bond distances and angles fall within the range for similar complexes: Farrar *et al.*, 1995; Dyson *et al.*, 2004; Cloete *et al.*, 2010; Engelbrecht *et al.*, 2010**a*,b*. Diphosphinoamine (PNP) and other P donor ligands (Keat *et al.*, 1981; Purcell *et al.*, 1995; Cotton *et al.*, 1996; Otto & Roodt, 2001; Fei *et al.*, 2003; Otto *et al.*, 2005; Muller *et al.*, 2008; Engelbrecht *et al.*, 2010**c*,d*;2011) with various substituents on both the P and N atoms form part of ongoing research in different catalytic olefin transformation reactions such as hydroformylation (Haumann *et al.*, 2004; Crous *et al.*, 2005), metathesis (Booyens *et al.*, 2007) and methoxycarbonylation (Ferreira *et al.*, 2007).

The title compound (Figure 1) crystallizes with two trifluoromethanesulfonate anions with the Pt^II^ atom situated on an inversion centre. The square-planar geometry around the metal centre is severely distorted as illustrated by the P1–Pt–P2 angle of 70.33 (4) °. The coordinated P1—N1—P2 angle indicates a severe distortion from the ideal trigonal-planar angle expected at the *sp*^2^-hybridized nitrogen. The P1–N1–P2 angle of the un-coordinated ligand of 121.76 (9) ° decreases to 102.3 (2) ° to accommodate coordination to the platinum. The N atom is displaced by 0.114 (4) Å from the C1, P1, P2 plane, while the Pt atom is perfectly planar with the phosphorous atoms. The orientation of the phenyl rings changes from a C*_s_* conformation when un-coordinated to a C*_2v_* conformation in the solid state in order to coordinated to the platinum.

For the coordinated ligand, the cyclopentane ring is twisted [*q*_2_ = 0.420 (5) Å, φ = 26.5 (8) °] (Cremer & Pople, 1975) with atom C2 deviating 0.645 (5) Å from the plane of the remaining four atoms. As for the un-coordinated ligand, (Engelbrecht *et al.*, 2010*d*) the cyclopentane ring is in an envelope conformation [*q*_2_ = 0.398 (2) Å, φ = 78.5 (3) °] with C3A as flap, which lies 0.590 (2) Å from the plane of the remaining four atoms. The disordered component of the cyclopentane ring also has an envelope conformation [*q*_2_ = 0.379 (6) Å, φ = 319.7 (10) °] with C5B as flap, lying -0.528 (2) Å from the plane of the other four atoms.

The trifluoromethanesulfonate anions are disordered over two positions with site occupancy factors of 0.511 (11):0.489 (11). The crystal packing is influenced by inter- and intra-molecular hydrogen bonds (Figure 2, Table 1).

### Experimental

[Pt(cod)Cl_2_] (20 mg, 0.0535 mmol) (cod = 1,5-cyclooctadiene) dissolved in the minimum amount of dichloromethane was added in a rapid drop-wise manner to a solution of bis(diphenylphosphino)cyclopentylamine (50.87 mg, 0.112 mmol) and silvertriflate (27.5 mg, 0.107 mmol) dissolved in the minimum volume of dichloromethane-methanol (1:1). After stirring for 20 min, the solvent was removed completely under reduced pressure. Dichloromethane was added until no further dissolution of solid was evident. The resulting heterogeneous mixture was filtered through celite to remove the insoluble AgCl by-product. The colourless solid product was precipitated upon addition of methanol followed by a reduction in solvent volume under reduced pressure. The compound was isolated by filtration and washed with diethyl ether (10 cm^3^). Layering of a dichloromethane solution of the product with methanol gave colourless crystals, suitable for X-ray diffraction. (Yield: 60 mg, 74%)

### Refinement

The methine, methylene and aromatic H atoms were placed in geometrically idealized positions at C—H = 1.00, 0.99 and 0.95 Å, respectively and constrained to ride on their parent atoms, with *U*_iso_(H) = 1.2*U*_eq_(C). The highest peak is located 0.00 Å from Pt1 and the deepest hole is situated 0.06 Å from P2. The residual electron-density features are probably a consequence of an imperfect absorption correction. The only way to secure a stable refinement with regards to the disordered anion was by adopting some atoms as isotropic. A series of EADP was used for neighbouring atoms and *DFIX* was applied in some cases to ensure stable refinement.

### Crystal data

**Table:**

[Pt(C_29_H_29_NP_2_)_2_](CF_3_SO_3_)_2_	*F*(000) = 1408
*M**_r_* = 1400.18	*D*_x_ = 1.624 Mg m^−^^3^
Monoclinic, *P*2_1_/*c*	Mo *K*α radiation, λ = 0.71073 Å
Hall symbol: -P 2ybc	Cell parameters from 9870 reflections
*a* = 10.041 (5) Å	θ = 2.8–28.1°
*b* = 13.662 (4) Å	µ = 2.71 mm^−^^1^
*c* = 20.928 (5) Å	*T* = 100 K
β = 93.916 (5)°	Cuboid, colourless
*V* = 2864.2 (19) Å^3^	0.19 × 0.18 × 0.16 mm
*Z* = 2	

### Data collection

**Table:**

Bruker APEXII CCD diffractometer	4869 reflections with *I* > 2σ(*I*)
Graphite monochromator	*R*_int_ = 0.101
φ and ω scans	θ_max_ = 28°, θ_min_ = 2.0°
Absorption correction: multi-scan (*SADABS*; Bruker, 2008)	*h* = −12→13
*T*_min_ = 0.627, *T*_max_ = 0.671	*k* = −18→18
44370 measured reflections	*l* = −27→27
6894 independent reflections	

### Refinement

**Table:**

Refinement on *F*^2^	Primary atom site location: structure-invariant direct methods
Least-squares matrix: full	Secondary atom site location: difference Fourier map
*R*[*F*^2^ > 2σ(*F*^2^)] = 0.040	Hydrogen site location: inferred from neighbouring sites
*wR*(*F*^2^) = 0.093	H-atom parameters constrained
*S* = 1.02	*w* = 1/[σ^2^(*F*_o_^2^) + (0.0387*P*)^2^ + 0.8274*P*] where *P* = (*F*_o_^2^ + 2*F*_c_^2^)/3
6894 reflections	(Δ/σ)_max_ = 0.001
398 parameters	Δρ_max_ = 1.39 e Å^−^^3^
7 restraints	Δρ_min_ = −1.42 e Å^−^^3^

### Special details

**Table:**

Experimental. The intensity data were collected on a Bruker X8 ApexII 4 K Kappa CCD diffractometer using an exposure time of 20 s/frame. A total of 1880 frames were collected with a frame width of 0.5° covering up to θ = 28.0° with 99.8% completeness accomplished.Spectroscopy data: ^1^H NMR (600 MHz, CD_2_Cl_2_): δ = 1.0 (m, 4H), 1.1 (m, 4H), 1.2 (m, 4H), 1.4 (m, 4H), 3.5 (m, 2H), 7.4 – 7.8 (m, 40H). ^31^P NMR (243 MHz, CD_2_Cl_2_): δ = 39.7 (t, ^1^J_Pt—P_ = 1063.0 Hz).
Geometry. All s.u.'s (except the s.u. in the dihedral angle between two l.s. planes) are estimated using the full covariance matrix. The cell s.u.'s are taken into account individually in the estimation of s.u.'s in distances, angles and torsion angles; correlations between s.u.'s in cell parameters are only used when they are defined by crystal symmetry. An approximate (isotropic) treatment of cell s.u.'s is used for estimating s.u.'s involving l.s. planes.
Refinement. Refinement of *F*^2^ against ALL reflections. The weighted *R*-factor *wR* and goodness of fit *S* are based on *F*^2^, conventional *R*-factors *R* are based on *F*, with *F* set to zero for negative *F*^2^. The threshold expression of *F*^2^ > 2σ(*F*^2^) is used only for calculating *R*-factors(gt) *etc*. and is not relevant to the choice of reflections for refinement. *R*-factors based on *F*^2^ are statistically about twice as large as those based on *F*, and *R*- factors based on ALL data will be even larger.

### Fractional atomic coordinates and isotropic or equivalent isotropic displacement parameters (Å^2^)

**Table:**

	*x*	*y*	*z*	*U*_iso_*/*U*_eq_	Occ. (<1)
C1	1.0762 (4)	0.3164 (3)	0.9779 (2)	0.0230 (10)	
H1	1.0169	0.3487	1.0081	0.028*	
C2	1.2188 (5)	0.3463 (3)	0.9988 (2)	0.0306 (11)	
H2A	1.2846	0.309	0.9754	0.037*	
H2B	1.238	0.3371	1.0455	0.037*	
C3	1.2184 (6)	0.4555 (4)	0.9805 (2)	0.0382 (12)	
H3A	1.1792	0.4957	1.0138	0.046*	
H3B	1.3102	0.4789	0.9748	0.046*	
C4	1.1334 (6)	0.4606 (4)	0.9179 (3)	0.0395 (13)	
H4A	1.1909	0.4654	0.8814	0.047*	
H4B	1.0748	0.5189	0.9174	0.047*	
C5	1.0489 (5)	0.3669 (3)	0.9123 (2)	0.0261 (10)	
H5A	1.0769	0.3247	0.8772	0.031*	
H5B	0.953	0.3827	0.9044	0.031*	
C11	1.2563 (4)	0.1091 (3)	0.91477 (19)	0.0207 (9)	
C12	1.3335 (5)	0.0312 (3)	0.9382 (2)	0.0264 (10)	
H12	1.2961	−0.017	0.9644	0.032*	
C13	1.4666 (5)	0.0238 (4)	0.9232 (2)	0.0330 (12)	
H13	1.5186	−0.0311	0.9374	0.04*	
C14	1.5222 (5)	0.0964 (4)	0.8878 (2)	0.0297 (11)	
H14	1.6139	0.0925	0.8795	0.036*	
C15	1.4480 (5)	0.1736 (4)	0.8645 (2)	0.0316 (11)	
H15	1.4876	0.2229	0.8401	0.038*	
C16	1.3142 (5)	0.1796 (3)	0.8768 (2)	0.0264 (10)	
H16	1.2613	0.232	0.8593	0.032*	
C21	0.9925 (4)	0.1201 (3)	0.85355 (19)	0.0201 (9)	
C22	1.0380 (4)	0.0582 (3)	0.8056 (2)	0.0236 (10)	
H22	1.1194	0.0232	0.8127	0.028*	
C23	0.9622 (5)	0.0492 (4)	0.7479 (2)	0.0272 (10)	
H23	0.9911	0.0073	0.7153	0.033*	
C24	0.8444 (5)	0.1013 (3)	0.7378 (2)	0.0265 (10)	
H24	0.7926	0.0946	0.6983	0.032*	
C25	0.8016 (5)	0.1625 (3)	0.7842 (2)	0.0263 (10)	
H25	0.7215	0.1989	0.7763	0.032*	
C26	0.8746 (4)	0.1713 (3)	0.8424 (2)	0.0222 (10)	
H26	0.8436	0.2127	0.8747	0.027*	
C31	1.1077 (4)	0.1741 (3)	1.11284 (19)	0.0208 (9)	
C32	1.2084 (5)	0.1038 (3)	1.1270 (2)	0.0263 (10)	
H32	1.2098	0.0452	1.1026	0.032*	
C33	1.3044 (5)	0.1194 (4)	1.1758 (2)	0.0317 (11)	
H33	1.3713	0.0716	1.1857	0.038*	
C34	1.3027 (5)	0.2060 (4)	1.2107 (2)	0.0346 (12)	
H34	1.37	0.2173	1.244	0.042*	
C35	1.2052 (5)	0.2756 (4)	1.1978 (2)	0.0364 (12)	
H35	1.2053	0.3344	1.2221	0.044*	
C36	1.1069 (5)	0.2591 (4)	1.1491 (2)	0.0302 (11)	
H36	1.0386	0.3063	1.1405	0.036*	
C41	0.8337 (4)	0.2049 (3)	1.0621 (2)	0.0209 (9)	
C42	0.7533 (5)	0.2500 (3)	1.0139 (2)	0.0267 (10)	
H42	0.7872	0.2625	0.9734	0.032*	
C43	0.6244 (5)	0.2764 (4)	1.0250 (2)	0.0324 (11)	
H43	0.57	0.3077	0.9922	0.039*	
C44	0.5735 (5)	0.2576 (4)	1.0840 (2)	0.0326 (12)	
H44	0.4837	0.2742	1.0909	0.039*	
C45	0.6538 (5)	0.2148 (4)	1.1327 (2)	0.0306 (11)	
H45	0.6198	0.2039	1.1733	0.037*	
C46	0.7842 (5)	0.1876 (3)	1.1222 (2)	0.0262 (10)	
H46	0.8392	0.1577	1.1554	0.031*	
N1	1.0451 (3)	0.2102 (3)	0.97884 (16)	0.0190 (8)	
P1	1.08226 (11)	0.11427 (8)	0.93121 (5)	0.01603 (7)	
P2	0.99258 (11)	0.15296 (8)	1.04487 (5)	0.01603 (7)	
Pt1	1	0	1	0.01603 (7)	
O1A	0.6874 (3)	0.3547 (3)	0.87585 (18)	0.0410 (9)	0.511 (11)
S1A	0.6839 (7)	0.4342 (5)	0.8293 (4)	0.0353 (13)	0.511 (11)
O2A	0.7720 (9)	0.4555 (9)	0.7931 (7)	0.080 (5)	0.511 (11)
O3A	0.5823 (9)	0.3523 (6)	0.7743 (3)	0.045 (3)	0.511 (11)
C01A	0.5385 (9)	0.4916 (7)	0.8440 (3)	0.0353 (13)	0.511 (11)
F3A	0.5273 (8)	0.5233 (6)	0.9030 (4)	0.048 (2)	0.511 (11)
F2A	0.4267 (8)	0.4383 (6)	0.8272 (6)	0.070 (3)	0.511 (11)
F1A	0.5064 (7)	0.5707 (5)	0.8087 (4)	0.042 (2)*	0.511 (11)
O1B	0.6874 (3)	0.3547 (3)	0.87585 (18)	0.0410 (9)	0.489 (11)
F3B	0.6075 (16)	0.5606 (9)	0.8840 (7)	0.129 (7)	0.489 (11)
O2B	0.7866 (7)	0.5037 (6)	0.8490 (5)	0.047 (3)	0.489 (11)
C01B	0.5446 (10)	0.5042 (8)	0.8351 (5)	0.0308 (12)	0.489 (11)
O3B	0.6795 (15)	0.4142 (10)	0.7618 (4)	0.082 (5)	0.489 (11)
S1B	0.6660 (6)	0.4067 (5)	0.8193 (4)	0.0308 (12)	0.489 (11)
F2B	0.4419 (11)	0.4684 (10)	0.8634 (6)	0.100 (5)	0.489 (11)
F1B	0.5215 (9)	0.5642 (6)	0.7862 (5)	0.058 (3)*	0.489 (11)

### Atomic displacement parameters (Å^2^)

**Table:**

	*U*^11^	*U*^22^	*U*^33^	*U*^12^	*U*^13^	*U*^23^
C1	0.027 (2)	0.019 (2)	0.023 (2)	0.0027 (19)	0.0023 (19)	0.0019 (19)
C2	0.034 (3)	0.028 (3)	0.029 (2)	−0.005 (2)	−0.002 (2)	−0.002 (2)
C3	0.047 (3)	0.032 (3)	0.036 (3)	−0.013 (3)	0.000 (2)	0.003 (2)
C4	0.050 (3)	0.028 (3)	0.039 (3)	−0.006 (3)	0.001 (3)	0.008 (2)
C5	0.031 (3)	0.023 (3)	0.024 (2)	0.003 (2)	0.000 (2)	0.0031 (19)
C11	0.024 (2)	0.021 (2)	0.017 (2)	0.0018 (18)	0.0011 (18)	−0.0008 (18)
C12	0.024 (2)	0.028 (3)	0.028 (2)	−0.0023 (19)	−0.001 (2)	0.000 (2)
C13	0.022 (2)	0.038 (3)	0.038 (3)	0.008 (2)	−0.003 (2)	0.002 (2)
C14	0.016 (2)	0.039 (3)	0.034 (3)	−0.002 (2)	0.004 (2)	−0.003 (2)
C15	0.027 (3)	0.038 (3)	0.029 (3)	0.000 (2)	0.004 (2)	0.002 (2)
C16	0.027 (3)	0.026 (3)	0.026 (2)	0.006 (2)	0.002 (2)	−0.001 (2)
C21	0.025 (2)	0.019 (2)	0.016 (2)	−0.0026 (18)	0.0029 (18)	0.0013 (17)
C22	0.024 (2)	0.024 (3)	0.023 (2)	0.0024 (19)	0.0018 (19)	−0.0010 (19)
C23	0.032 (3)	0.028 (3)	0.022 (2)	−0.002 (2)	0.002 (2)	−0.003 (2)
C24	0.028 (3)	0.030 (3)	0.021 (2)	−0.002 (2)	−0.0043 (19)	0.001 (2)
C25	0.023 (2)	0.026 (3)	0.030 (2)	0.003 (2)	0.001 (2)	0.002 (2)
C26	0.024 (2)	0.019 (2)	0.023 (2)	0.0034 (19)	0.0017 (19)	−0.0007 (18)
C31	0.026 (2)	0.019 (2)	0.017 (2)	0.0012 (18)	0.0005 (18)	0.0003 (18)
C32	0.029 (3)	0.025 (3)	0.024 (2)	0.000 (2)	0.000 (2)	−0.006 (2)
C33	0.027 (3)	0.041 (3)	0.026 (2)	0.005 (2)	−0.003 (2)	−0.002 (2)
C34	0.036 (3)	0.038 (3)	0.029 (3)	−0.001 (2)	−0.007 (2)	−0.007 (2)
C35	0.047 (3)	0.034 (3)	0.027 (3)	−0.004 (3)	−0.003 (2)	−0.012 (2)
C36	0.038 (3)	0.028 (3)	0.025 (2)	0.003 (2)	−0.001 (2)	−0.003 (2)
C41	0.023 (2)	0.019 (2)	0.021 (2)	0.0018 (18)	0.0023 (18)	−0.0035 (18)
C42	0.030 (3)	0.026 (3)	0.025 (2)	0.004 (2)	0.006 (2)	0.000 (2)
C43	0.033 (3)	0.032 (3)	0.032 (3)	0.009 (2)	0.001 (2)	0.004 (2)
C44	0.025 (3)	0.034 (3)	0.039 (3)	0.007 (2)	0.008 (2)	−0.005 (2)
C45	0.034 (3)	0.029 (3)	0.029 (2)	0.001 (2)	0.008 (2)	−0.004 (2)
C46	0.032 (3)	0.022 (2)	0.025 (2)	0.007 (2)	0.006 (2)	−0.001 (2)
N1	0.0225 (19)	0.020 (2)	0.0147 (16)	0.0061 (15)	0.0041 (15)	0.0005 (15)
P1	0.01943 (12)	0.01522 (12)	0.01339 (11)	0.00270 (10)	0.00077 (7)	0.00016 (10)
P2	0.01943 (12)	0.01522 (12)	0.01339 (11)	0.00270 (10)	0.00077 (7)	0.00016 (10)
Pt1	0.01943 (12)	0.01522 (12)	0.01339 (11)	0.00270 (10)	0.00077 (7)	0.00016 (10)
O1A	0.037 (2)	0.035 (2)	0.051 (2)	0.0101 (17)	0.0047 (18)	0.0085 (18)
S1A	0.049 (3)	0.025 (3)	0.032 (2)	0.023 (2)	0.0062 (19)	0.0012 (18)
O2A	0.039 (5)	0.096 (9)	0.106 (12)	−0.009 (6)	0.023 (6)	0.052 (8)
O3A	0.060 (6)	0.042 (5)	0.032 (4)	0.011 (4)	−0.010 (4)	−0.024 (4)
C01A	0.049 (3)	0.025 (3)	0.032 (2)	0.023 (2)	0.0062 (19)	0.0012 (18)
F3A	0.044 (5)	0.051 (5)	0.050 (4)	0.015 (3)	0.018 (4)	−0.008 (4)
F2A	0.028 (4)	0.051 (5)	0.127 (9)	−0.004 (3)	−0.032 (5)	−0.003 (6)
O1B	0.037 (2)	0.035 (2)	0.051 (2)	0.0101 (17)	0.0047 (18)	0.0085 (18)
F3B	0.169 (15)	0.075 (9)	0.129 (11)	0.060 (9)	−0.091 (11)	−0.077 (8)
O2B	0.020 (4)	0.042 (5)	0.080 (7)	0.001 (4)	0.011 (4)	0.018 (5)
C01B	0.0310 (18)	0.029 (3)	0.033 (3)	0.0079 (18)	0.0072 (15)	0.0028 (19)
O3B	0.115 (13)	0.099 (10)	0.031 (5)	0.050 (10)	−0.002 (6)	−0.028 (6)
S1B	0.0310 (18)	0.029 (3)	0.033 (3)	0.0079 (18)	0.0072 (15)	0.0028 (19)
F2B	0.077 (9)	0.161 (14)	0.064 (7)	0.058 (9)	0.026 (7)	0.002 (7)

### Geometric parameters (Å, º)

**Table:**

C1—N1	1.484 (6)	C32—C33	1.372 (6)
C1—C2	1.524 (6)	C32—H32	0.95
C1—C5	1.543 (6)	C33—C34	1.390 (7)
C1—H1	1	C33—H33	0.95
C2—C3	1.540 (7)	C34—C35	1.378 (7)
C2—H2A	0.99	C34—H34	0.95
C2—H2B	0.99	C35—C36	1.389 (6)
C3—C4	1.516 (7)	C35—H35	0.95
C3—H3A	0.99	C36—H36	0.95
C3—H3B	0.99	C41—C42	1.391 (6)
C4—C5	1.535 (7)	C41—C46	1.403 (6)
C4—H4A	0.99	C41—P2	1.805 (4)
C4—H4B	0.99	C42—C43	1.379 (6)
C5—H5A	0.99	C42—H42	0.95
C5—H5B	0.99	C43—C44	1.391 (7)
C11—C12	1.387 (6)	C43—H43	0.95
C11—C16	1.400 (6)	C44—C45	1.385 (7)
C11—P1	1.805 (4)	C44—H44	0.95
C12—C13	1.397 (7)	C45—C46	1.393 (6)
C12—H12	0.95	C45—H45	0.95
C13—C14	1.379 (7)	C46—H46	0.95
C13—H13	0.95	N1—P2	1.703 (4)
C14—C15	1.362 (7)	N1—P1	1.704 (4)
C14—H14	0.95	P1—Pt1	2.3139 (12)
C15—C16	1.388 (6)	P1—P2	2.6534 (15)
C15—H15	0.95	P2—Pt1	2.2943 (13)
C16—H16	0.95	Pt1—P2^i^	2.2943 (13)
C21—C26	1.381 (6)	Pt1—P1^i^	2.3139 (12)
C21—C22	1.413 (6)	O1A—S1A	1.458 (9)
C21—P1	1.806 (4)	S1A—O2A	1.237 (12)
C22—C23	1.388 (6)	S1A—C01A	1.704 (2)
C22—H22	0.95	S1A—O3A	1.859 (11)
C23—C24	1.384 (6)	C01A—F3A	1.320 (2)
C23—H23	0.95	C01A—F1A	1.336 (5)
C24—C25	1.373 (6)	C01A—F2A	1.364 (13)
C24—H24	0.95	F3B—C01B	1.398 (15)
C25—C26	1.384 (6)	O2B—S1B	1.873 (12)
C25—H25	0.95	C01B—F2B	1.318 (2)
C26—H26	0.95	C01B—F1B	1.320 (5)
C31—C36	1.387 (6)	C01B—S1B	1.850 (12)
C31—C32	1.412 (6)	O3B—S1B	1.226 (12)
C31—P2	1.794 (4)		
			
N1—C1—C2	116.9 (4)	C35—C34—C33	121.2 (5)
N1—C1—C5	115.1 (3)	C35—C34—H34	119.4
C2—C1—C5	104.0 (4)	C33—C34—H34	119.4
N1—C1—H1	106.7	C34—C35—C36	119.4 (5)
C2—C1—H1	106.7	C34—C35—H35	120.3
C5—C1—H1	106.7	C36—C35—H35	120.3
C1—C2—C3	101.6 (4)	C31—C36—C35	120.5 (5)
C1—C2—H2A	111.4	C31—C36—H36	119.8
C3—C2—H2A	111.4	C35—C36—H36	119.8
C1—C2—H2B	111.4	C42—C41—C46	120.0 (4)
C3—C2—H2B	111.4	C42—C41—P2	120.4 (3)
H2A—C2—H2B	109.3	C46—C41—P2	119.0 (3)
C4—C3—C2	104.6 (4)	C43—C42—C41	119.9 (4)
C4—C3—H3A	110.8	C43—C42—H42	120
C2—C3—H3A	110.8	C41—C42—H42	120
C4—C3—H3B	110.8	C42—C43—C44	120.5 (4)
C2—C3—H3B	110.8	C42—C43—H43	119.8
H3A—C3—H3B	108.9	C44—C43—H43	119.8
C3—C4—C5	107.6 (4)	C45—C44—C43	120.0 (4)
C3—C4—H4A	110.2	C45—C44—H44	120
C5—C4—H4A	110.2	C43—C44—H44	120
C3—C4—H4B	110.2	C44—C45—C46	120.2 (5)
C5—C4—H4B	110.2	C44—C45—H45	119.9
H4A—C4—H4B	108.5	C46—C45—H45	119.9
C4—C5—C1	104.0 (4)	C45—C46—C41	119.4 (4)
C4—C5—H5A	111	C45—C46—H46	120.3
C1—C5—H5A	111	C41—C46—H46	120.3
C4—C5—H5B	111	C1—N1—P2	122.5 (3)
C1—C5—H5B	111	C1—N1—P1	133.6 (3)
H5A—C5—H5B	109	P2—N1—P1	102.32 (19)
C12—C11—C16	119.0 (4)	N1—P1—C11	113.27 (19)
C12—C11—P1	119.2 (3)	N1—P1—C21	112.14 (19)
C16—C11—P1	121.7 (3)	C11—P1—C21	105.09 (19)
C11—C12—C13	119.7 (5)	N1—P1—Pt1	93.07 (13)
C11—C12—H12	120.1	C11—P1—Pt1	118.91 (14)
C13—C12—H12	120.1	C21—P1—Pt1	114.36 (14)
C14—C13—C12	119.9 (5)	C11—P1—P2	124.81 (14)
C14—C13—H13	120.1	C21—P1—P2	128.24 (15)
C12—C13—H13	120.1	Pt1—P1—P2	54.50 (4)
C15—C14—C13	121.1 (4)	N1—P2—C31	110.5 (2)
C15—C14—H14	119.4	N1—P2—C41	107.96 (19)
C13—C14—H14	119.4	C31—P2—C41	108.0 (2)
C14—C15—C16	119.5 (5)	N1—P2—Pt1	93.78 (13)
C14—C15—H15	120.2	C31—P2—Pt1	115.73 (15)
C16—C15—H15	120.2	C41—P2—Pt1	119.61 (15)
C15—C16—C11	120.6 (4)	C31—P2—P1	120.20 (15)
C15—C16—H16	119.7	C41—P2—P1	128.04 (14)
C11—C16—H16	119.7	Pt1—P2—P1	55.19 (3)
C26—C21—C22	119.9 (4)	P2^i^—Pt1—P2	180.0000 (10)
C26—C21—P1	123.0 (3)	P2^i^—Pt1—P1^i^	70.31 (4)
C22—C21—P1	116.6 (3)	P2—Pt1—P1^i^	109.69 (4)
C23—C22—C21	119.2 (4)	P2^i^—Pt1—P1	109.69 (4)
C23—C22—H22	120.4	P2—Pt1—P1	70.31 (4)
C21—C22—H22	120.4	P1^i^—Pt1—P1	180.0000 (10)
C24—C23—C22	119.9 (4)	O2A—S1A—O1A	126.8 (7)
C24—C23—H23	120	O2A—S1A—C01A	131.2 (9)
C22—C23—H23	120	O1A—S1A—C01A	101.7 (5)
C25—C24—C23	120.7 (4)	O2A—S1A—O3A	98.5 (9)
C25—C24—H24	119.6	O1A—S1A—O3A	87.3 (5)
C23—C24—H24	119.6	C01A—S1A—O3A	87.0 (5)
C24—C25—C26	120.2 (4)	F3A—C01A—F1A	102.7 (8)
C24—C25—H25	119.9	F3A—C01A—F2A	107.2 (8)
C26—C25—H25	119.9	F1A—C01A—F2A	97.2 (7)
C21—C26—C25	120.1 (4)	F3A—C01A—S1A	116.7 (6)
C21—C26—H26	120	F1A—C01A—S1A	116.7 (7)
C25—C26—H26	120	F2A—C01A—S1A	114.1 (7)
C36—C31—C32	119.1 (4)	F2B—C01B—F1B	118.6 (10)
C36—C31—P2	122.7 (3)	F2B—C01B—F3B	101.7 (10)
C32—C31—P2	118.1 (3)	F1B—C01B—F3B	105.8 (11)
C33—C32—C31	120.5 (4)	F2B—C01B—S1B	111.0 (9)
C33—C32—H32	119.8	F1B—C01B—S1B	113.1 (7)
C31—C32—H32	119.8	F3B—C01B—S1B	104.9 (7)
C32—C33—C34	119.4 (5)	O3B—S1B—C01B	103.5 (7)
C32—C33—H33	120.3	O3B—S1B—O2B	98.8 (9)
C34—C33—H33	120.3	C01B—S1B—O2B	81.4 (6)
			
N1—C1—C2—C3	170.6 (4)	C1—N1—P2—C31	−54.4 (4)
C5—C1—C2—C3	42.5 (4)	P1—N1—P2—C31	113.0 (2)
C1—C2—C3—C4	−37.7 (5)	C1—N1—P2—C41	63.5 (4)
C2—C3—C4—C5	18.9 (6)	P1—N1—P2—C41	−129.1 (2)
C3—C4—C5—C1	7.2 (6)	C1—N1—P2—Pt1	−173.7 (3)
N1—C1—C5—C4	−160.2 (4)	P1—N1—P2—Pt1	−6.25 (16)
C2—C1—C5—C4	−31.0 (5)	C1—N1—P2—P1	−167.5 (4)
C16—C11—C12—C13	−0.6 (7)	C36—C31—P2—N1	81.5 (4)
P1—C11—C12—C13	176.9 (4)	C32—C31—P2—N1	−94.2 (4)
C11—C12—C13—C14	3.1 (7)	C36—C31—P2—C41	−36.4 (4)
C12—C13—C14—C15	−3.0 (8)	C32—C31—P2—C41	147.9 (3)
C13—C14—C15—C16	0.2 (7)	C36—C31—P2—Pt1	−173.6 (3)
C14—C15—C16—C11	2.4 (7)	C32—C31—P2—Pt1	10.8 (4)
C12—C11—C16—C15	−2.2 (7)	C36—C31—P2—P1	123.4 (4)
P1—C11—C16—C15	−179.6 (4)	C32—C31—P2—P1	−52.3 (4)
C26—C21—C22—C23	−0.6 (6)	C42—C41—P2—N1	23.8 (4)
P1—C21—C22—C23	171.0 (3)	C46—C41—P2—N1	−164.9 (4)
C21—C22—C23—C24	0.6 (7)	C42—C41—P2—C31	143.3 (4)
C22—C23—C24—C25	0.3 (7)	C46—C41—P2—C31	−45.4 (4)
C23—C24—C25—C26	−1.3 (7)	C42—C41—P2—Pt1	−81.5 (4)
C22—C21—C26—C25	−0.4 (6)	C46—C41—P2—Pt1	89.8 (4)
P1—C21—C26—C25	−171.4 (3)	C42—C41—P2—P1	−14.4 (5)
C24—C25—C26—C21	1.3 (7)	C46—C41—P2—P1	156.9 (3)
C36—C31—C32—C33	−0.2 (7)	C11—P1—P2—N1	84.5 (3)
P2—C31—C32—C33	175.7 (4)	C21—P1—P2—N1	−77.7 (3)
C31—C32—C33—C34	−1.0 (7)	Pt1—P1—P2—N1	−172.4 (2)
C32—C33—C34—C35	1.1 (8)	N1—P1—P2—C31	−85.7 (3)
C33—C34—C35—C36	0.0 (8)	C11—P1—P2—C31	−1.2 (2)
C32—C31—C36—C35	1.2 (7)	C21—P1—P2—C31	−163.3 (2)
P2—C31—C36—C35	−174.4 (4)	Pt1—P1—P2—C31	101.95 (17)
C34—C35—C36—C31	−1.1 (7)	N1—P1—P2—C41	69.7 (3)
C46—C41—C42—C43	−0.8 (7)	C11—P1—P2—C41	154.1 (3)
P2—C41—C42—C43	170.4 (4)	C21—P1—P2—C41	−8.0 (3)
C41—C42—C43—C44	−0.6 (7)	Pt1—P1—P2—C41	−102.71 (19)
C42—C43—C44—C45	2.0 (8)	N1—P1—P2—Pt1	172.4 (2)
C43—C44—C45—C46	−2.0 (8)	C11—P1—P2—Pt1	−103.14 (18)
C44—C45—C46—C41	0.6 (7)	C21—P1—P2—Pt1	94.75 (18)
C42—C41—C46—C45	0.8 (7)	N1—P2—Pt1—P1^i^	−175.23 (12)
P2—C41—C46—C45	−170.5 (4)	C31—P2—Pt1—P1^i^	69.81 (17)
C2—C1—N1—P2	88.8 (4)	C41—P2—Pt1—P1^i^	−62.09 (17)
C5—C1—N1—P2	−148.7 (3)	P1—P2—Pt1—P1^i^	180
C2—C1—N1—P1	−74.2 (5)	N1—P2—Pt1—P1	4.77 (12)
C5—C1—N1—P1	48.3 (6)	C31—P2—Pt1—P1	−110.19 (17)
C1—N1—P1—C11	48.2 (4)	C41—P2—Pt1—P1	117.91 (17)
P2—N1—P1—C11	−117.2 (2)	N1—P1—Pt1—P2^i^	175.24 (12)
C1—N1—P1—C21	−70.6 (4)	C11—P1—Pt1—P2^i^	−65.98 (16)
P2—N1—P1—C21	124.1 (2)	C21—P1—Pt1—P2^i^	59.23 (16)
C1—N1—P1—Pt1	171.5 (4)	P2—P1—Pt1—P2^i^	180
P2—N1—P1—Pt1	6.19 (16)	N1—P1—Pt1—P2	−4.76 (12)
C1—N1—P1—P2	165.3 (5)	C11—P1—Pt1—P2	114.02 (16)
C12—C11—P1—N1	114.3 (4)	C21—P1—Pt1—P2	−120.77 (16)
C16—C11—P1—N1	−68.3 (4)	O2A—S1A—C01A—F3A	−117.3 (14)
C12—C11—P1—C21	−122.9 (4)	O1A—S1A—C01A—F3A	57.3 (10)
C16—C11—P1—C21	54.5 (4)	O3A—S1A—C01A—F3A	143.9 (9)
C12—C11—P1—Pt1	6.6 (4)	O2A—S1A—C01A—F1A	4.5 (17)
C16—C11—P1—Pt1	−176.0 (3)	O1A—S1A—C01A—F1A	179.1 (8)
C12—C11—P1—P2	71.5 (4)	O3A—S1A—C01A—F1A	−94.3 (10)
C16—C11—P1—P2	−111.1 (3)	O2A—S1A—C01A—F2A	116.7 (15)
C26—C21—P1—N1	−23.0 (4)	O1A—S1A—C01A—F2A	−68.6 (7)
C22—C21—P1—N1	165.6 (3)	O3A—S1A—C01A—F2A	18.0 (9)
C26—C21—P1—C11	−146.5 (4)	F2B—C01B—S1B—O3B	126.0 (14)
C22—C21—P1—C11	42.2 (4)	F1B—C01B—S1B—O3B	−10.2 (14)
C26—C21—P1—Pt1	81.3 (4)	F3B—C01B—S1B—O3B	−125.0 (13)
C22—C21—P1—Pt1	−90.0 (3)	F2B—C01B—S1B—O2B	−137.0 (12)
C26—C21—P1—P2	18.4 (5)	F1B—C01B—S1B—O2B	86.9 (9)
C22—C21—P1—P2	−153.0 (3)	F3B—C01B—S1B—O2B	−27.9 (10)

Symmetry code: (i) −*x*+2, −*y*, −*z*+2.

### Hydrogen-bond geometry (Å, º)

**Table:**

*D*—H···*A*	*D*—H	H···*A*	*D*···*A*	*D*—H···*A*
C25—H25···O3*A*	0.95	2.52	3.398 (9)	154
C26—H26···O1*B*	0.95	2.5	3.238 (6)	135
C34—H34···O3*A*^ii^	0.95	2.38	3.127 (10)	135
C45—H45···O3*A*^iii^	0.95	2.3	3.229 (9)	165
C15—H15···O3*A*^iv^	0.95	2.47	3.420 (11)	178

Symmetry codes: (ii) *x*+1, −*y*+1/2, *z*+1/2; (iii) *x*, −*y*+1/2, *z*+1/2; (iv) *x*+1, *y*, *z*.

## References

[bb1] Booyens, S., Roodt, A. & Wendt, O. F. (2007). *J. Organomet. Chem.* **692**, 5508–5512.

[bb2] Brandenburg, K. & Putz, H. (2005). *DIAMOND* Crystal Impact GbR, Bonn, Germany.

[bb3] Bruker (2008). *SAINT-Plus* and *SADABS* Bruker AXS Inc., Madison, Wisconsin, USA.

[bb4] Bruker (2011). *APEX2* Bruker AXS Inc., Madison, Wisconsin, USA.

[bb5] Cloete, N., Visser, H. G. & Roodt, A. (2010). *Acta Cryst.* E**66**, m51–m52. 10.1107/S160053680905301X PMC2980280 21579949

[bb6] Cotton, F. A., Kuhn, F. E. & Yokochi, A. (1996). *Inorg. Chim. Acta*, **252**, 251–256.

[bb7] Cremer, D. & Pople, J. A. (1975). *J. Am. Chem. Soc.* **97**, 1354–1358.

[bb8] Crous, R., Datt, M., Foster, D., Bennie, L., Steenkamp, C., Huyser, J., Kirsten, L., Steyl, G. & Roodt, A. (2005). *Dalton Trans.* pp. 1108–1116. 10.1039/b416917d 15739014

[bb9] Dyson, P. J., Fei, Z. & Scopelliti, R. (2004). *Eur. J. Inorg. Chem.* pp. 530–537.

[bb10] Engelbrecht, I., Visser, H. G. & Roodt, A. (2010*a*). *Acta Cryst.* E**66**, m922–m923. 10.1107/S1600536810025560 PMC3007544 21588157

[bb11] Engelbrecht, I., Visser, H. G. & Roodt, A. (2010*b*). *Acta Cryst.* E**66**, m994–m995. 10.1107/S1600536810028795 PMC3007244 21588214

[bb12] Engelbrecht, I., Visser, H. G. & Roodt, A. (2010*c*). *Acta Cryst.* E**66**, o2881. 10.1107/S1600536810041711 PMC3009108 21589062

[bb13] Engelbrecht, I., Visser, H. G. & Roodt, A. (2010*d*). *Acta Cryst.* E**66**, o3322–o3323. 10.1107/S1600536810048907 PMC3011404 21589599

[bb14] Engelbrecht, I., Visser, H. G. & Roodt, A. (2011). *Acta Cryst.* E**67**, o2041–o2042. 10.1107/S1600536811027656 PMC3213490 22091069

[bb15] Farrar, D. G. & Browning, C. S. (1995). *J. Chem. Soc. Dalton Trans.* pp. 521–530.

[bb16] Farrugia, L. J. (1999). *J. Appl. Cryst.* **32**, 837–838.

[bb17] Fei, Z., Scopeleti, R. & Dyson, P. J. (2003). *Dalton Trans.* pp. 2772–2779.

[bb18] Ferreira, A. C., Crous, R., Bennie, L., Meij, A. M. M., Blann, K., Bezuidenhoudt, B. C. B., Young, D. A., Green, M. J. & Roodt, A. (2007). *Angew. Chem. Int. Ed.* **46**, 2273–2275. 10.1002/anie.200603751 17299818

[bb19] Haumann, M., Meijboom, R., Moss, J. R. & Roodt, A. (2004). *Dalton Trans.* pp. 1679–1686. 10.1039/b403033h 15252562

[bb20] Keat, R., Manojlovic-Muir, L., Muir, K. W. & Rycroft, D. S. (1981). *J. Chem. Soc. Dalton Trans.* pp. 2192–2198.

[bb21] Muller, A., Otto, S. & Roodt, A. (2008). *Dalton Trans.* pp. 650–657. 10.1039/b712782k 18217121

[bb22] Otto, S., Ionescu, A. & Roodt, A. (2005). *J. Organomet. Chem.* **690**, 4337–4342.

[bb23] Otto, S. & Roodt, A. (2001). *Inorg. Chem. Commun.* **4**, 49–52.

[bb24] Purcell, W., Basson, S. S., Leipoldt, J. G., Roodt, A. & Preston, H. (1995). *Inorg. Chim. Acta*, **234**, 153–156.

[bb25] Sheldrick, G. M. (2008). *Acta Cryst.* A**64**, 112–122. 10.1107/S0108767307043930 18156677

